# Dilatational-plasticity opens a new mechanistic pathway for macromolecular transport across glassy interfaces

**DOI:** 10.1038/s41598-024-66217-4

**Published:** 2024-08-09

**Authors:** Ajay Vallabh, Nikhil Padhye

**Affiliations:** Department of Mechanical Engineering, University of New Hampshire, Durham, USA

**Keywords:** Glasses, Coarse-grained models

## Abstract

Interdiffusion-based macromolecular transport across glassy interfaces is reportedly achieved at high temperatures in accordance with the classical model of reptation. Here, for the first time, we report a new mechanistic pathway for achieving solid-state glassy joining by triggering rapid macromolecular acceleration through mechanical deformation. Large-scale molecular simulations reveal that active plastic deformation in glassy polymers, at temperatures well below the bulk (and surface) glass transition temperatures $\text {T}_g^b$ (and $\text {T}_g^s$), causes segmental translations of macromolecules leading to interfacial interpenetrations, and the formation of new entanglements. The mechanistic basis for this new type of bonding is identified as molecular-scale dilatations and densifications during deformation-induced mobility. The reported insights open promising avenues for achieving quick, strong, and energetically less-intensive joining of polymeric glasses across various sectors.

## Introduction

Adhesion between polymeric interfaces plays a vital role in production of small-molecule therapeutics^1^, regenerative medicines with targeted systemic release^2,3^, fabrication of electronic displays^4^, biosensors, MEMS, microfluidics^5^, recyclable plastics, and smart textiles^6,7^. Several large-scale manufacturing processes such as 3D printing, extrusion, injection molding, calendering, thermoforming, etc., rely directly on polymer adhesion for successful product fabrication. Since the earliest discovery of polymer adhesion via interdiffusion^8^, it has been conventionally accepted that when two polymers are brought into contact at a temperature *above* the glass transition temperature, under moderate contact pressure, the polymer molecules interdiffuse and form entanglements across the interface over the experimental timescales to cause bonding through the formation of entanglements^9–16^. In recent unprecedented experimental studies^17,18^, researchers have reported that the macromolecular transport, and thereby polymer adhesion, can occur rapidly through mechanical deformation alone, without any heating, at temperatures well below $\text {T}_g$ (several 10s of degrees below $\text {T}_g$) of a glassy polymer. The mechanistic origins of this new type of deformation-induced bonding (DIB) in the solid state have remained elusive thus far and are reported in this letter.

The strength of the polymer interface during bonding in the melt state reportedly depends on healing time, temperature, contact pressure, molecular structure, and other chemical or physical characteristics of the polymer. The interdiffusion proceeds via reptation until the interface disappears^19^, and the interfacial toughness ($G_c$) and shear strength ($\sigma _s$) follow a time-dependent scaling of $t^{1/2}$ and $t^{1/4}$, respectively. Molecular simulations have also confirmed these bonding trends^20–22^. All such studies have emphasized that temperatures above $\text {T}_g$ are essential for polymer adhesion to take place. At temperatures well-below $\text {T}_g$, bonding between glassy polymers due to interdiffusion cannot be detected on experimental timescales because the timescales for relaxation in the glassy state are extremely large^23–26^, and the system is effectively frozen concerning any cooperative segmental motions ($\alpha -$relaxation)^27,28^ to yield entanglements across the interface. Despite frozen mobilities at temperatures below $\text {T}_g$, some researchers have claimed that polymer bonding can occur via reptation dynamics at temperatures below the bulk-$\text {T}_g$^29,30^, over timescales of hours, by arguing that the surface of a glassy polymer has a lowered glass transition temperature ($\text {T}_g^s$) compared to that of the bulk polymer ($\text {T}_g^b$), and a noticeable molecular-scale diffusivity exists on the polymer free-surface that enables interfacial interdiffusion and bonding. These studies, however, have completely ignored the role of mechanical activation due to application of pressure, which can cause creep and enhanced molecular mobility^31^, and thereby bonding. In contrast, the recent experimental solid-state DIB studies^17^ revealed that polymer films with varying molecular weights could be bonded in the time on the order of a fraction of a second, with bonding strengths correlating directly with the gross plastic strain, and all polymer blends achieved similar levels of bonding strengths in the same interval of time. These distinct characteristics of DIB led to the conclusion that the mechanism of macromolecular transport across the interface during deformation-induced bonding should necessarily be different from the reptation-based interdiffusion that occurs in a melt state; since, in reptation-based interdiffusion, the diffusion coefficient shows a strong molecular weight dependence, whereas in solid-state DIB, different polymer films with varying molecular weights were bonded in the same time interval to similar bonding strengths. Additionally, the bond strengths in DIB exhibited a non-monotonic correlation with the imposed plastic strain, whereas in reptation-based healing the interfacial strengths grow monotonically with respect to time until the interface is fully healed.

**Figure 1:**
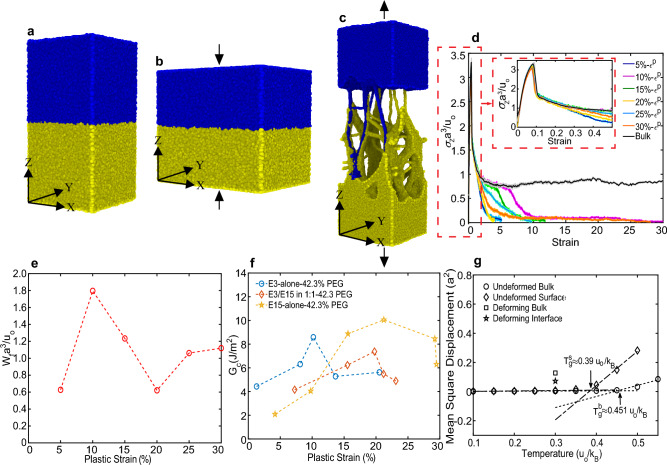
Deformation-induced bonding of polymer samples under plane strain compression. (**a–c**) Represent states of two polymer samples before bonding, being subject to compressive plastic straining, and debonding during uniaxial tension, respectively. (**d**) Debonding stress–strain response of bulk samples bonded at 5%, 10%, 15%, 20%, 25%, and 30% plastic strains. $X\%$-$\epsilon ^p$ abbreviates the imposed bonding plastic strain. The thin lines are raw stress–strain data, and the thick lines represent smoothed data using a moving average filter. (**e**) Debonding work of fracture $W_f$ (non-dimensionalized with $u_o/a^3$) versus plastic strain plot. The $W_f$ values are based on the area under the stress–strain curves obtained from MD simulations. (**f**) Fracture toughness $G_c (\text{J/m}^2)$ versus plastic strain plots for E3/E15 in 1:1–42.3% polyethylene glycol (PEG), E3-alone-42.3% polyethylene glycol (PEG), and E15-alone-42.3% polyethylene glycol (PEG) taken from Ref.^17^. (**g**) Estimation of surface and bulk glass transition temperatures $T_g^s$ and $T_g^b$, respectively, by calculating MSD as a function of temperature.

Using large-scale molecular simulations, we discover that compressive plastic deformation of glassy polymers, held in intimate contact, triggers requisite macromolecular acceleration, and opens a new mechanistic pathway through molecular-scale dilatations (and densifications), that facilitates chain interpenetrations across the interfaces to cause interfacial bonding. Solid-state glassy samples of sizes $66.5 \times 66.5 \times 66.5a^{3}$ (*a* denoting the bead size), at a temperature T $=0.3u_o/k_B$, were constructed using coarse-grained Kremer–Grest (K–G) model (well-known to represent linear amorphous polymers^20–22,32^ phenomenologically), with $\text {T}^b_g \approx 0.45\text{u}_o/\text{k}_B$, by quenching the equilibrated molecular melts (see “Methods” section). (Also, see Supplementary Sect. 1 for detailed explanations for the choice of coarse-grained bead-spring model.) Free surfaces of the two samples were brought into molecular contact at $Z=0$ plane, and subject to plane-strain active plastic deformation up to different levels of compressive plastic strains (5%, 10%, 15%, 20%, 25%, and 30%), Fig. 1a, b. (see Supplementary Video 1). NPT conditions were maintained during compression. To evaluate the interfacial strengths, the newly bonded interfaces were subject to uniaxial tensile loading, Fig. 1c, and the strengths of the bonded samples were characterized by the debonding stress–strain curves, Fig. 1d. Corresponding to different levels of bonding strains, the debonding tensile tests exhibited a narrow elastic region, with maximum elastic stress around 3.4$\sigma _z a^3/u_o$, after which yielding and strain hardening characteristics were noted. The debonding tests revealed the events of chain pull-outs and scissions (see Supplementary Video 2), thereby providing the molecular evidence that active plastic deformation during compression was sufficient to trigger enhanced molecular mobility and chain interpenetration across the interfaces to yield bonding below the bulk-$\text {T}_g$. The area under the debonding stress-strain curve was chosen as the quantitative measure for the work of fracture ($\text {W}_f$) and has units of $u_o/a^3$, Fig. 1e. $\text {W}_f$ correlated non-monotonically with respect to the imposed plastic strain, showing consistency with the experimental results^17^, Fig. 1f (see Supplementary Sect. 5 for discussions regarding bonding strengths obtained through DIB compared to bulk strengths). Postprocessing of the interfacial entanglements revealed that macromolecular reorientation in the direction of principal stretches at higher plastic strains caused non-monotonic growth in the number of entanglements across the interface, thereby leading to non-monotonic correlations between the bonding strengths and the imposed plastic strains (see Supplementary Sect. 4).

Glass transition temperatures of the free surface ($\text {T}^s_g$) and the interior bulk ($\text {T}^b_g$) of the polymer samples were estimated to be $\text {T}^s_g=0.39 u_o/k_B$ and $\text {T}^b_g=0.45 u_o/k_B$, respectively, Fig. 1g. The naturally existent molecular mobility, characterized in terms of average mean-squared-displacements (MSDs) at the free surfaces, averaged over time intervals of $50\tau$ in sets of thin layers within the bulk and at the interface, without deformation at T$=0.3 u_o/k_B$ (the temperature at which molecular DIB experiments were carried out), was found to be negligible compared to the mobility at the free surfaces, without deformation at $\text {T}^s_g=0.39u_o/k_B$, indicating that the free surface of the polymer is in a true glassy state at T $=0.3 u_o/k_B$, and that the molecular-activity at the free surfaces at T $=0.3 u_o/k_B$ is similar to those of frozen glassy bulks (see “Methods” section and Supplementary Sect. 2 for calculations of $\text {T}^s_g$, $\text {T}^b_g$, and MSDs). Thus, it is clear that even though $\text {T}^s_g < T^b_g$, the absence of long-range mobility at the free surfaces at T $=0.3 u_{o}/k_{B}$, in the absence of deformation cannot cause bonding when two pieces of a glassy polymer are brought in molecular proximity. During active plastic deformation (in 0–30% deformation range at T $=0.3 u_{o}/k_{B}$), MSDs of polymer beads were calculated and found to be enhanced by an order to two orders in magnitude when compared to the undeformed sample at T $=0.3 u_o/k_B$ (see Supplementary Sect. 2).

Previous studies^33,34^ have shown that the free volume in a glassy polymer is distributed over a broad range of molecular dimensions and that this distribution is responsible for the phenomenon of viscoelasticity, diffuse shear transformations, and accelerated polymer mobility during deformation^35^. Furthermore, local liquid-like environments percolate through the glassy volume and enable rapid segmental relaxations. An actively deforming glass is identified as a heterogeneously dilated continuum with molecular mobility comparable to that at above $\text {T}_g$^36,37^. In context to bonding, although macromolecular acceleration is a requirement for a polymer molecule to migrate across the interface quickly, it is not a sufficient condition to cause interpenetration because an accelerated molecule near the interface can incrementally translate parallel to the bonding plane without causing any interpenetration. The classical notions of shear transformation zones (STZs), acting as carriers of plastic deformation, do not provide any molecular-scale information about the polymer dynamics during deformation. Thus, in order to identify the mechanistic origins of DIB, we analyzed the following: evolution of the microstructure during deformation, segmental-level volume changes, interfacial segmental rearrangements, and segmental transport across the interfaces. A local state variable entitled local number density ($\rho _N$) was defined for each polymer bead (subsequently also referred to as the molecular site), with $\rho _N$ representing the number of neighboring beads in a spherical volume ($V_l$) of radius $r_l=2.5a$ centered at any particular bead. Since the LJ potential cut-off radius is 2.5*a*, $\rho _N$ quantitatively captures the local packing density at any molecular site. The average densities of the solid glass at T $=0.3 u_{o}/k_{B}$ and the polymer melt at T $=0.5u_{o}/k_{B}$ were found to be $\rho _g=1.003a^{-3}$ and $\rho _l = 0.95a^{-3}$, respectively, and accordingly the *average* local number density for the glassy and the melt states were determined to be ${\rho }_{N_g}$ $=65.65$ and ${\rho }_{N_l}=62.17$, respectively. Another metric called the radial distribution function (RDF)^38,39^ was used to characterize the dynamics of the microstructure during deformation (see “Methods” section, Supplementary Sect. 4). RDF represents the probability *g*(*r*) of finding a bead that is at a distance *r* from any reference bead, and thus captures the aggregated global information about the distribution of beads within the microstructure.

**Figure 2:**
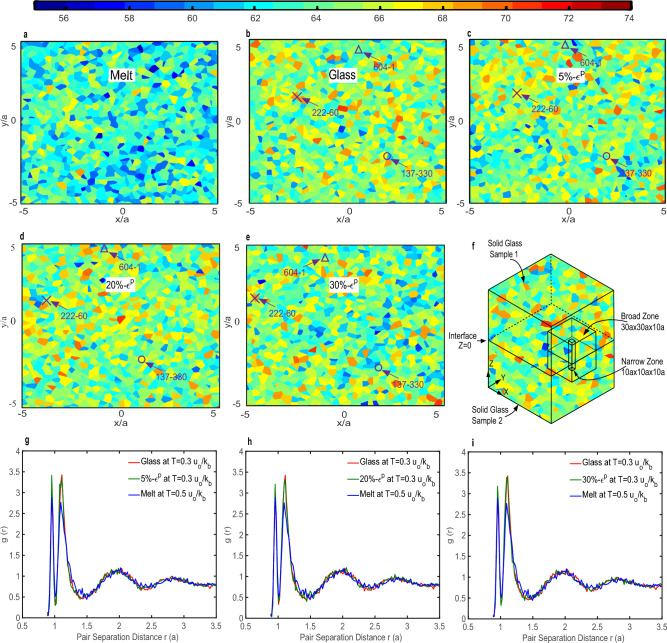
Local number density and radial distribution function of small $10a \times 10a \times 10a$ across the interface. (**a**) Local number density distribution in a polymer melt equilibrated *T* = $0.5u_{o}/k_{B}$. (**b**) Quenched glass *T* = $0.3u_o/k_B$ . (**c–e**) Samples deformed at 5%, 20% & 30% at *T* = $0.3u_o/k_B$, respectively. (**f**) Region in which local number density distribution is calculated. (**g–i**) Plots of radial distribution function (RDF) comparing DIB bonded samples at 5%, 20% & 30%, with undeformed glass and the polymer melt.

Figure 2a–e show the spatial distribution of $\rho _N$ in the equilibrated melt at T=$0.5u_{o}/k_{B}$, quenched glass at T=$0.3u_{o}/k_{B}$, and glasses that underwent 5%, 20% & 30% plastic deformation at T=$0.3u_{o}/k_{B}$, respectively, in a $10a \times 10a \times 10a$ sized narrow zone at the interface (see Fig. 2f). Figure 2g–i show the corresponding plots of RDFs for 5%, 20% & 30% deformed samples in comparison with the RDFs of the quenched glass at T=$0.3u_{o}/k_{B}$, and the equilibrated melt at T=$0.5u_{o}/k_{B}$. The $\rho _N$ plot of the melt state, Fig. 2a, is dominated by the blue regions (indicative of loose molecular packing) and is in clear contrast with respect to the glassy states (with or without deformation, Fig. 2b–e). The quenched and deformed glasses (Fig. 2b–e) were found to be microstructurally heterogeneous, with local liquid-like packets embedded within the solid-state bulk. As the deformation proceeded from 0 to 30% plastic strain, liquid-like regions appeared and disappeared at different molecular sites. As such, there was no evidence of increased liquidity, i.e., the appearance of more “blue regions”, in the deforming glasses compared to the undeformed glass, but it was evidently clear that the local liquid-like regions were moving through the glassy bulk during deformation. A particular molecular site that was densely packed at a particular instant quickly transformed into a liquid-like region and vice-versa, and these transformations provided the spatial allowances for molecular rearrangements to occur. These local liquid-like regions aided the cooperative relaxations of the cluster of beads during incremental straining as the global deformation followed kinematic compatibility due to externally imposed loads at the boundary.

The RDFs in Fig. 2g–i revealed two sharp peaks followed by a diffused pattern. The first and second peaks corresponded to the distances at which finite extensible nonlinear elastic (FENE) potential (the intra-particle pair potential) and the Lennard–Jones (LJ) potential (the inter-particle pair potential) reached their minimum values, respectively. Across all these plots, the RDF curves of various deformed and undeformed glasses were similar, indicating that the distribution of the molecular distances, on an average, within the microstructure was *not* altered during deformation. The first and second peaks were, however, wider and lower for the melts in comparison to the glasses, indicating a loosely packed fluid-like behavior. We conclude that the plastic deformation in itself does not transform a solid-state glass into a melt state (thermodynamically speaking), and that the macroscopic behavior of glass during deformation at the constant temperature is still solid; however, plastic deformation triggers heterogeneous evolution of microstructure, and liquid-like regions of mobility within the glassy structure percolate throughout the bulk during deformation. The molecular-scale dimension of size 2.5*a* is the relevant length scale for capturing these local structural changes. To understand the nature of the molecular-scale mobility in deforming glasses, motions of several randomly chosen beads were followed. For the sake of illustration here, we selected beads with IDs 604-1, 222-60, and 137-330 (see Supplementary Sect. 4 for bead ID nomenclature), which were initially present in the “narrow region” in the glassy state (see the marked molecular sites in Fig. 2b, and definition of the “narrow region” in Fig. 2f). During deformation, the locations of these beads changed dramatically (see the location of the marked beads at different plastic strains in Fig. 2c–e, and compare these displaced positions with those in the undeformed glass in Fig. 2b).

**Figure 3:**
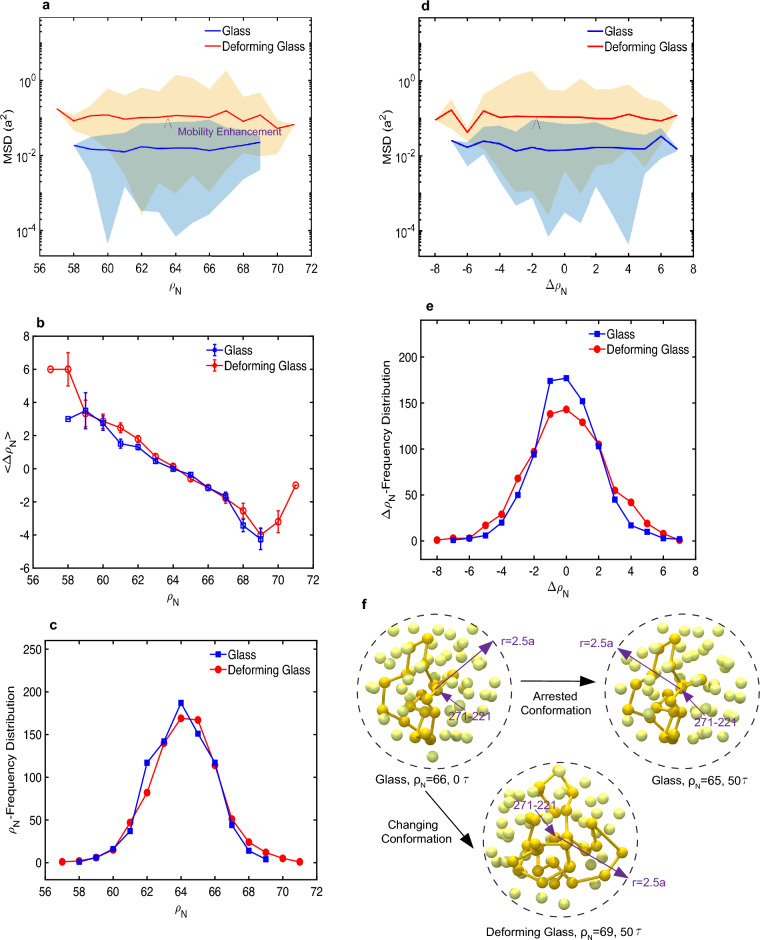
Chain-end-motion. (**a, d**) Comparison of chain-end displacements with respect to local number density, and gradient in local number density. (**b**) The correlation between gradient in local number density and local number density. (**c**) The frequency distribution of local number density. (**e**) The frequency distribution of gradient in local number density. (**f**) Conformation arrest and transformation in the glassy state and deforming glass, respectively.

To quantify and correlate the accelerated molecular mobility during plastic deformation with the changes in the microstructure and contrast it with the arrested long-range mobility in the glassy state, we computed the MSDs of the chain-end- and non-chain-end-beads, both in the glassy state and during deformation. The data for MSDs of chain-end-beads with respect to the time-dependent local number density ($\rho _N$) and temporal gradient of the local number density (denoted by $\triangle \rho _N$) are shown in Fig. 3a, d. Similar characteristic trends were also found for non-chain-end-beads (see Supplementary Fig. 11). From these plots, it is clear that deformation causes at least an order to two orders of magnitude enhancement in the molecular mobility over the entire range of $\rho _N$, and $\triangle \rho _N$. The most notable feature of these MSD curves is that the quantitative values for MSDs in the deforming glass are comparable to the dimension *a* (i.e., the bead size), implying that sufficiently large molecular-level openings can be created on one side of the interface through which a bead of size *a* can interpenetrate and yield bonding. The plots of $<\triangle \rho _N>$ vs $\rho _N$, both for the glassy state and deforming glass, are shown in Fig. 3b. The negative correlation between $\rho _N$ and $\triangle \rho _N$ reveals that the soft sites (low $\rho _N$ values) undergo densification (positive $\triangle \rho _N$), whereas the hard sites (high $\rho _N$ values) dilate (negative $\triangle \rho _N$). Although molecular-scale dilatations and densifications also occur in the undeformed glass, they are not accompanied by an enhancement in molecular mobility. After observing the exhaustive data, both for the deforming and undeformed glasses, we found that the number of dilatations was approximately equal to the number of densifications (see Supplementary Sect. 4 for the sample data), which is consistent with no noticeable changes in the RDF plots between the deforming and undeformed glasses. The frequency distributions of $\triangle \rho$, both for the deforming and undeformed glasses, are shown in Fig. 3e, where we note that the glassy state is characterized by a higher number of non-volume changing events than the deforming glasses. In the deforming glass, the number of events corresponding to larger-size dilatations or densifications has increased. A similar behavior is noted for the frequency distribution of $\rho _N$, in Fig. 3c, i.e., the occurrences of relatively harder or softer sites (marked by extreme values of $\rho _N$) are more prominent in deforming glasses. Figure 3f shows an example where, despite molecular-scale dilatations in the glassy state, the conformation of polymer chains is essentially arrested. In contrast, the conformations are noticeably altered in the deforming glass. In the glassy state, without deformation, the conformations of the polymer chains are found to be essentially frozen and the observed molecular-scale dilatations (or densifications) essentially correspond to molecular-scale vibrations, which are non-zero even at the solid-state temperatures (below $T_g^b$ or $T_g^s$).

**Figure 4:**
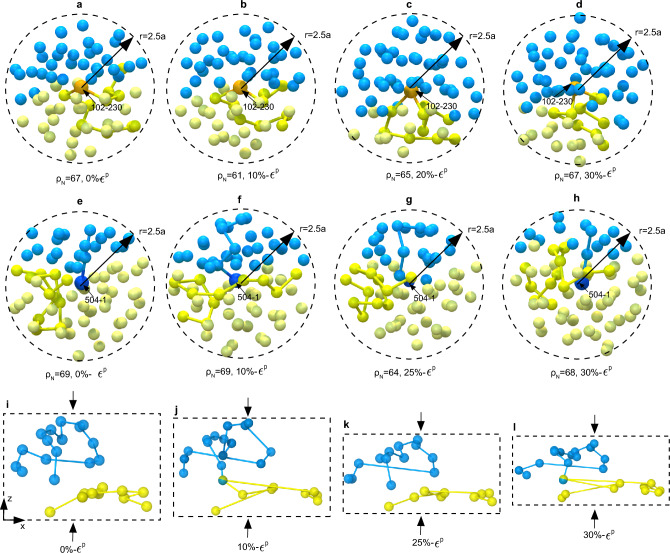
Molecular interpenetration, formation, disengagement, and reformation of entanglements. (**a–d**) A non-chain-end with ID 102-230, at different stages of plastic strains 0–30% with corresponding $\rho _N$, that exhibits interpenetration motion across the interface. (**e–h**) A chain-end with ID 504-1, at different stages of plastic strains 0–30% with corresponding $\rho _N$, that also exhibits interpenetration across the interface. (**i–l**) Demonstrate primitive paths for opposite side chains (ID 504 & ID 53) at 0, 10, 25, and 30% plastic strains, respectively. Each blue and yellow beads represent topological constraints.

The topological constraints restrict the dynamics of a polymer chain in an entangled melt, and the motion of a polymer chain occurs along the contours of the tube in which it is trapped (via reptation). Therefore, the chain-ends play a leading role during interdiffusion across the interface, causing interpenetration and the formation of new interfacial entanglements. In the case of deforming solid-state glassy networks, like in polymer melts, the polymer chains cannot cross each other, nor can the polymer segments break their topological bonds and displace arbitrarily; however, unlike polymer melts without deformation, favorable molecular-scale dilatations (or densifications) in conjunction with enhanced mobility can provide opportunities for, both, the chain-ends and non-chain-ends to interpenetrate across the interface to yield bonding. This is precisely the mechanistic difference between DIB and conventional interdiffusion-based bonding in polymer melts. Figure 4a–d illustrates how a polymer chain (ID 102), with a particular non-chain-end (ID 102-230) located near the interface at 0% plastic strain, interpenetrated from the lower sample into the upper sample during 20% plastic strain deformation. Following a spherical region of influence of radius 2.5*a*, it was found that neighboring beads in this region are displaced by new neighboring beads upon straining; the dilatation-plasticity under compressive traction causes cooperative relaxation of the cluster of beads surrounding the non-chain-end-bead, and motion of the marked polymer segment enables relative slippage (or sliding) causing the non-chain-end to penetrate into the other side of the interface (see Supplementary Video 3). This mechanism of molecular interpenetration is infeasible in an equilibrated molecular melt without deformation since the polymer segments are constrained to lie within their tubes. Another mode of interpenetration and subsequent entanglement formation is shown in Fig. 4e–h, where a chain-end (ID 504-1) of a polymer chain (ID 504) translates from the upper sample into the lower sample. In this case, the chain-end interpenetration was followed by the formation of a new entanglement, subsequent disengagement, and reformation. Figure 4i–l show primitive paths of two interfacial chains (ID 504 & ID 53) at 0%, 10%, 25%, & 30% deformation, respectively. A primitive path^40^ is a geometrically constructed shortest path between the end-points of a particular polymer chain in which the chain’s contour can relax without crossing any obstacle. Each surrounding bead between the two ends of the chain along the primitive path represents a topological constraint, where the polymer chain forms entanglements with the surrounding chains. As the deformation proceeded, the randomly coiled chain configurations were compressed, and the chain segments were preferentially oriented in the direction of principal stretches. Whenever favorable molecular conditions emerged, the chain-end interpenetrated from the upper sample into the bottom sample and formed an entanglement. We confirmed the formation of the entanglements using Z1-code^41–43^ (see “Methods” section for entanglement calculations). Continued straining caused the disengagement of this entanglement and subsequent reformation (See Supplementary Video 4). These behaviors explain the non-monotonic bonding trends in DIB as well.

Although the disappearance of the interface and mixing of homopolymeric chains from opposite sides is favorable, both energetically and entropically, we emphasize that once a glassy interface is subject to deformation, and the kinetically trapped glass is activated, not every bit of incremental deformation, and resulting segmental motion needs to cause interpenetration. The site of molecular-scale dilatation or densification and incremental molecular motion must be compatible in a geometric sense to result in an interpenetration. The dependence of this interpenetration upon molecular parameters (composition, molecular weight, physical and chemical properties) and processing conditions remains an open question at this stage and requires further investigation. The reptation timescales for the studied molecular systems were estimated to be $$3M\tau$$^22^, whereas the total deformation (for 0–30% plastic strain) and bonding experiments were achieved in timescales on the order of $$1951\tau$$, highlighting a striking contrast. According to the conventional interdiffusion theories, bonding between thermoplastics in solid-state (well below $$\text {T}_g$$) on experimental timescales is infeasible (implying that thermally unstable polymeric materials cannot be bonded through heat-induced interdiffusion), high molecular weight ($$\text {M}_w$$) polymers in melt state will require an exorbitantly long time for interdiffusion, and interfaces will not acquire bulk material strength until interdiffusion is complete (up to the reptation timescales), immiscible polymers will not yield interdiffusion bonding and polymers with high melting temperatures will require excessive heating^1^. Using DIB we can bond glassy polymers below $$\text {T}_g$$, quickly, with bonding strengths approaching the bulk-like strength from below (see Supplementary Sect. 4). Thus, DIB has opened new pathways by overcoming barriers the conventional reptation-based model sets forth. Molecular engineering of new materials, interfacial states, and processes that can exploit the dilatational-plasticity for achieving quick and tailored adhesion hold great promise in enabling new applications. For example, the current market for small-molecule pharmaceuticals in the U.S. is approximately $600 billion/year (with 25% costs associated with manufacturing). Downstream continuous manufacturing of pharmaceuticals, relying on low-temperature bonding of biothermo-plastics, can contribute to these savings significantly and meet global medicine demands^1,44^. Other examples of manufacturing sectors in the US that heavily rely on polymer bonding are laser plastic welding (on course to reach a market value of US$1.5 billion by 2025), adhesives (estimated to be $15.0 billion in 2020), injection molding (current market size of US$265.1 billion), 3D printing (currently valued at US$520.5 million with an expected compound annual growth rate (CAGR) of 23.7% from 2020 to 2027), and biodegradable or compostable plastics (estimated at US$3.27 Bn in 2019 and is expected to expand at a revenue-based CAGR of 9.4%), to name a few^1^. To enable new and efficient ways of manufacturing plastic-based products involving bonding in all these areas, that consume less energy, assure product quality, and are environmentally friendly, the role of DIB is expected to be critical.

## Methods

### MD simulations

Large-scale Atomic/Molecular Massively Parallel Simulator (LAMMPS) from Sandia National Laboratories was used to carry out the molecular simulations on NH BioMade cluster. The bonding experiments were carried out in four primary steps (a) equilibration of polymer melts, (b) formation of quenched glasses, (c) plastic compression of two polymer glasses held in molecular proximity, and (d) debonding tensile tests. See further details in Supplementary Sect. 1. Kremer–Grest (K–G) model was used to model polymer chains. For LJ potential a cut-off radius $r_c$ of $2^{(1/6)}a$ and 2.5*a* was chosen during initial melt preparation, and subsequent deformation, respectively (see Supplementary Sect. 1 for more details). For FENE the cut-off radius and spring stiffness were chosen as $R_o=1.5a$ and $k=30u_oa^{-2}$, respectively. Each polymer sample was sized as $66.5 \times 66.5 \times 66.5a^{3}$ and contained $M=500$ polymer chains, with each chain comprising $N=500$ coarse-grained beads. The equilibration step was performed under NPT conditions with $P=0$ at $T=1.0u_{o}/k_{B}$. The equilibrated melt sample was quenched below to a solid-state glass at $T=0.3u_{o}/k_{B}$. Quenching rate of $\dot{T}=2 \times 10^{-3} u_{o}/k_{B}\tau$ was used. Plane strain compressive plastic deformation was carried out between two polymer samples in the range of 0% to 30% plastic strain, where two halves were compressed by a rigid ramp at a velocity of approximately 0.01 *a*/$\tau$. During the debonding tensile tests, the two halves of the bonded sample were separated with a constant velocity $v=2 \times 0.005 a\tau ^{-1}$ leading to nominal strain-rate of $2 \times 10^{-4}\tau ^{-1}$, and Quartic potential was used to model bond-breaking with parameter values of $K=2351u_{o}/k_{B}$, $B=-0.7425a$, $R_c=1.5a$, and $U_o=92.74467u_o$. All NVT and NPT simulations were performed using the Nose-Hoover thermostat and Nose-Hoover barostat. Newton’s equation of motion was integrated using a velocity-Verlet algorithm with a time step $\delta t\le 0.01\tau$. 1 million $\tau$ is abbreviated as $1M\tau$.

### Calculation of surface and bulk glass transition temperatures

The surface and bulk glass transition temperatures were calculated according to a standard procedure proposed in the literature^45^. Starting from a temperature of $T=1.0u_{o}/k_{B}$ the system was cooled down to $0.1u_{o}/k_{B}$ at decrements of $0.05u_{o}/k_{B}$. At each temperature, the system was first relaxed for $10,000\tau$, and then simulated for $10,000\tau$ under NPT conditions. During this period, MSDs were calculated in sets of thin layers, at the free surface, and within the bulk, over intervals of $50\tau$. The inflection points on the MSD vs. temperature plots were identified as the glass transition points. An alternate method of estimating glass transition temperature was also deployed, and gave similar results (see Supplementary Sect. 2).

### Calculation of $\triangle Z$ and *MSD* of chain-bead to characterize molecular mobility

The position vectors of the beads were extracted at increments of $0.5\tau$ during the 0–30% deformation period. Corresponding values of $\rho _N$ and $\triangle \rho _N$ were also calculated at these time intervals. The z-coordinate was utilized to compute incremental displacement $\triangle Z$ along the Z-axis, and used in the computation of mean absolute z-displacement $<|\triangle Z|>$. Similarly, the incremental square displacement vector $\triangle r^2$ was used to compute the MSDs. See Supplementary Sect. 3 for complete details.

### Calculation of entanglements

Entanglements due to topological constraints imposed by surrounding chains were identified using the Z1 algorithm^41–43^. Z1 algorithm is based on a geometrical approach that determines entanglements directly without referring to statistics of the primitive path. This unique approach speeds up this algorithm several orders of magnitude compared to PPA^46^ (see Supplementary Sect. 6).

### Supplementary Information

Supplementary Information.

Supplementary Video 1.

Supplementary Video 2.

Supplementary Video 3.

Supplementary Video 4.

## Data Availability

The datasets used and/or analysed during the current study available from the corresponding author on reasonable request.
