# Supplementary material for: Dilatational-plasticity opens a new mechanistic pathway for macromolecular transport across glassy interfaces
Source: Sci Rep. 2024 Aug 9;14:18552. doi: 10.1038/s41598-024-66217-4 (PMC11315893; doi:10.1038/s41598-024-66217-4)
Supplement: Supplementary file 1 — Supplementary Information. [file 41598_2024_66217_MOESM1_ESM.pdf]

Supplementary Information: Dilatational-Plasticity Opens a New Mechanistic Pathway for Macromolecular Transport Across Polymeric Interfaces Yielding Solid-State Bonding

1 MOLECULAR DYNAMICS SIMULATIONS: EQUILIBRATION, QUENCHING, BONDING, AND DEBONDING

Coarse-grained (CG) models are computationally cheaper, capable of approaching larger spatial and temporal scales (compared to, e.g., all-explicit atomistic models), and retain the essential chain-like nature of the molecules that cannot cross each other. CG-based molecular dynamics methods have been successfully applied in modeling the dynamics of diffusion in polymer melts[8, 10], bonding through interdiffusion in polymer melts and debonding failure between polymer interfaces[2–6], and mechanical behavior of glassy polymers while capturing the effects of strain rate, temperature, molecular entanglement density, and quenching rate[7]. With the correct choice of intra- and inter-molecular potentials, the coarse-grained models can produce a range of mechanical responses such as softening, strain hardening, and anisotropy. Although coarse-grained models neglect the chemical details, scaling trends with respect to chain lengths, and intrinsic chain dynamics are expected to be universal in glassy polymers, therefore, they are deemed as an appropriate tool for studying the phenomenological behavior of deformation-induced bonding.

The polymer chains were represented using the Kremer-Grest (K-G) model[8]. In the K-G model, monomers are represented as beads, and are connected with each other through springs along the chain backbone. The beads interact with a truncated and shifted Lenard-Jones (LJ) potential $U_{LJ}(r)$ as

$$U_{LJ}(r) = 4u_o \left[\left(\frac{a}{r}\right)^{12} - \left(\frac{a}{r}\right)^{6} - \left(\frac{a}{r_c}\right)^{12} + \left(\frac{a}{r_c}\right)^{6} \right],$$

where r_c is the cut-off radius chosen as $2^{1/6}a$ for the initial polymer melt preparation, and after equilibiration set to $2.5a$ (following the methodology presented in[5, 8]), a is the bead size, u_o is the binding energy, m is the mass of single bead, and τ is the characteristic timescale given as $\tau = a\,(m/u_o)^{\frac{1}{2}}$. In our LAMMPS simulations, all the quantities were expressed in LJ units. The interaction between topologically connected beads of a single chain were modeled using an unbreakable finitely extensible non-linear elastic (FENE) potential

$$U_{FENE}(r) = -\frac{1}{2}kR_o^2 ln \left[1 - \left(\frac{r}{R_o}\right)^{2} \right],$$

where R_o and k are the equilibrium bond length, and bond coefficient, respectively, for the beads. R_o and k were taken as $1.5a$, and $30u_oa^{-2}$, respectively. LJ and FENE potentials were used during equilibration, quenching, and deformation-induced bonding simulations. During debonding, in uniaxial testing, the FENE potential was replaced with a Quartic potential[13],

$$U_Q(r) = K(r - R_c)^2(r - R_c)(r - R_c - B) + U_o,$$

which allowed for simulating bond-breaking between the beads as the chains were stretched. The Quartic potential parameters were chosen as $K = 2351u_o/k_B$, $B = -0.7425a$, $R_c = 1.5a$, and $U_o = 92.74467u_o$. These parameters ensured that the equilibrium bond length based on $U_Q(r)$ was same as that for U_{FENE}[5].

Polymer samples of size $66.5 \times 66.5 \times 66.5a^3$ were constructed using a standard method from the literature[1]. Each sample comprised $M = 500$ polymer chains, with each chain containing $N = 500$ beads. Periodic boundary conditions were used in all directions during the initial sample generation. The chains were then unwrapped in the Z-direction, rigid walls were imposed on top and bottom surfaces, and chains were compressed to produce the desired size initial box. This sample was equilibrated at $T = 1.0u_o/k_B$, under NPT conditions, with fixed boundary conditions in the Z-direction (imposed by repulsive confining walls). The sample pressure was

maintained at $P = 0$ by allowing expansion or contraction along X-direction, with damping pressure coefficient $P_{damp} = 1000 * dt$. The equilibrated melt sample was quenched to a temperature $T = 0.3u_o/k_B$, at a quenching rate of $\dot{T} = 2 \times 10^{-3} u_o/k_B\tau$, below the glass transition temperature (which was estimated to be $T_g = 0.45u_o/k_B$), to produce a solid-state glass. Pressure $P = 0$ was maintained, and NPT ensemble, with damping pressure coefficient $P_{damp} = 1000 * dt$ for P_{xx} and P_{yy}, was used. The quenched sample was re-equilibriated (for $0.1M\tau$) to eliminate any residual stresses. Density of the quenched glass was found to be $\rho \approx 1.003a^{-3}$ (consistent with what is expected in a solid-state glass). To perform bonding experiments, the free surfaces of the two glass samples were first brought into molecular proximity without overlap at $Z = 0$. The repulsive walls, introduced during equilibration, were removed at the common interface such that free surfaces came into molecular proximity under Van der Waals forces. The two glasses were then compressed by moving the top and bottom walls along the Z-axis in the opposite directions. Plane strain conditions were maintained in the Y-direction, and due to compression in the Z-direction, the sample expanded along the X-direction. Once the sample assembly was deformed to a desired level of compression, it was equilibrated for $0.1M\tau$ to ensure elastic recovery. Different bonded samples, corresponding to different levels of imposed plastic strain, were prepared. Figure 1 shows a sample stress-strain curve during compression bonding, which is consistent with solid-state glassy behavior.

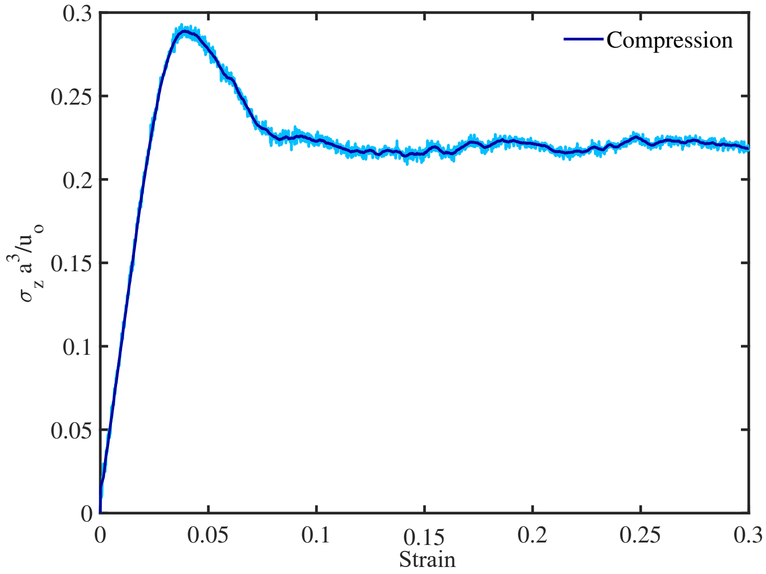

Figure 1. Stress-strain response corresponding to compression bonding between two polymer samples.

Debonding experiments were performed by subjecting the bonded sample to uniaxial tensile loading. A region of length $L_z^o = 50a$, centered around the bonded interface in the bonded assembly, was selected for the debonding tensile test. Material regions outside this central region were rigidly held and displaced in opposite directions each at a constant velocity $v = 0.005a\tau^{-1}$ along the Z-axis. The corresponding stress-strain curves, based on true stress and nominal strain measures, were collected. The tensile tests were carried under NVT conditions, and the temperature was maintained at $T = 0.3u_o/k_B$ by using a damping temperature rate $T_{damp} = 100 * dt$.

2 CALCULATION OF SURFACE AND BULK GLASS TRANSITION TEMPERATURES

Two approaches from the literature, based on the calculation of free volume and mean-square-displacement (MSD), were used to compute the glass-transition temperatures. The free volume approach is a classical procedure for computing the glass transition temperature of the polymer bulk, whereas the MSD approach has gained popularity for estimating the glass transition temperature on the polymer free surface[9]. MSD approach can also be used to estimate the bulk glass transition temperatures. In both these approaches, a melt sample of size $66.5 \times 66.5 \times 66.5a^3$ was prepared according to the procedure described in Section 1, with periodic boundary conditions in all directions. The sample comprised $M = 500$ polymer chains, with each chain containing $N = 500$ beads, and parametric values for LJ and FENE potential chosen same as those listed in Sec-

tion 1. The sample was equilibrated at a temperature T= $1.0u_o/k_B$, and pressure $P = 0$. Further details for both these approaches are given as follows.

(a) Free volume approach: After a well-equilibrated melt sample was prepared, it was quenched from a temperature $T = 1.0u_o/k_B$ to $T = 0.1u_o/k_B$, at a quenching rate of $\dot{T} = 2 \times 10^{-3}u_o/(k_B\tau)$, and constant pressure $P = 0$. An NPT ensemble with pressure damping coefficient $P_{damp} = 1000 * dt$ was used during the quenching. The sample was allowed to expand or contract in all directions. Specific volume (averaged over the entire sample) with respect to temperature was collected at an interval of 1τ during the quenching. Figure 2 shows the specific volume vs temperature plot. The inflection point on this plot gave an estimate for the bulk glass transition temperature as 0.45 u_o/k_B.

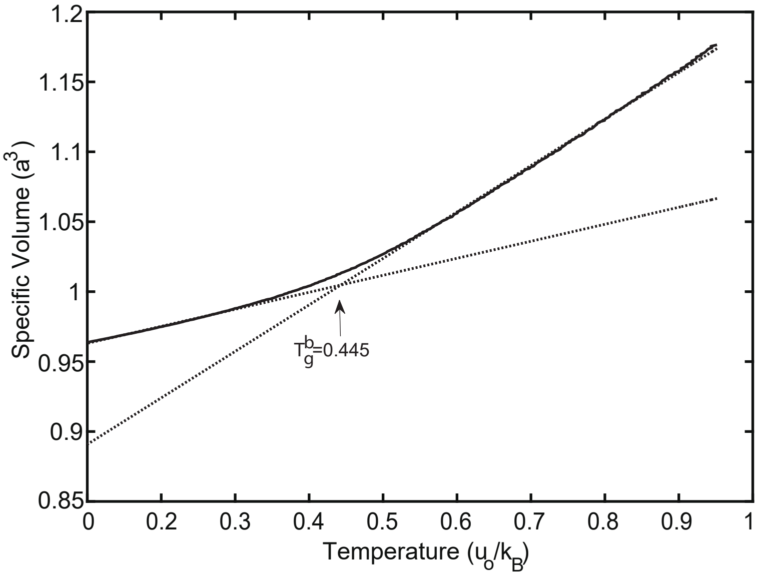

Figure 2. Free volume approach to compute the bulk glass transition temperature ${T_g}^b$. ${T_g}^b$ is estimated to be $0.445u_o/k_B$.

(b) MSD approach: In this approach[9], the glass transition temperatures at the surface and the bulk were determined by evaluating the mean square displacements (MSD) of the polymer segments in the surface and bulk regions, respectively, as a function of temperature. We first started with the same well-equilibrated melt sample with non-periodic boundary conditions in Z-direction, as described earlier, and then the sample was quenched in multiple stages (quasi-static manner) from a temperature $T = 1.0u_o/k_B$ to $T = 0.1u_o/k_B$, at a quenching rate $\dot{T} = 2 \times 10^{-3}u_o/(k_B\tau)$, and constant pressure $P = 0$. An NPT ensemble was chosen, with damping temperature and pressure coefficients as $T_{damp} = 100 * dt$, and $P_{damp} = 1000 * dt$, respectively. During quenching the sample was allowed to expand or contract in X- and Y-directions. At every $0.05u_o/k_B$ unit reduction in temperature, the system was relaxed for 10000τ, and then simulated for 10000τ for collection of the mobility data. To collect this mobility data, we divided the polymer sample into multiple layers along the Z-axis, with each layer of thickness a units. Three layers were chosen, both, at the surface and in the bulk, and position vectors of the polymer segments in these layers were documented at increments of 50τ. Accordingly, mean square displacements (MSDs), averaged over all segments in the respective surface and bulk layers, were computed. The inflexion point in the MSD vs temperature plot, both, in surface and bulk layers led to the estimation of surface and bulk glass transition temperatures, T_g^s and T_g^b, respectively. Figure 3 shows variation of surface and bulk MSD measurements as a function of the temperature.

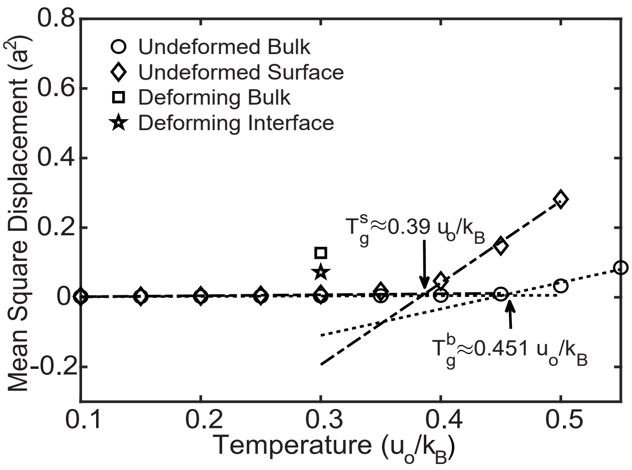

Figure 3. MSD approach to compute the bulk ($T_g{}^b$) and surface ($T_g{}^s$) glass transition temperatures.

We compared the bulk glass transition temperatures according to both approaches and found an excellent match. The quantitative data for MSD at different temperatures in the bulk and surface layers is given in the first two columns of Table 1. Clearly, the MSD decreases as the temperature is decreased. We also computed the mobility data for the deforming glass at $T = 0.3u_o/k_B$, over an interval of 50τ (the value of 50τ was chosen from the literature[9]), and found MSD to be $0.1275184a^2$ and $0.0720257a^2$ in bulk and interfacial layers, respectively. The MSD values in undeforming glass at $T = 0.3u_o/k_B$ in bulk and surface layers were found to be $0.003595854a^2$ and $0.00814626a^2$, respectively. These data show two orders and one order enhancement in molecular mobility in bulk and surface regions, respectively, in the deforming glass compared to the undeformed glass.

Table 1. The MSD data for estimating bulk and surface glass transition temperatures. MSD data in deforming glass is also listed in the fourth column.

**Temperature** (u_o/k_B)	**MSD in the bulk layer** (a^2)	**MSD in the surface layer** (a^2)	**MSD during** 0-30% **deformation at** T = $0.3u_o/k_b$
0.1	0.00178002	0.00236983	
0.15	0.001921507	0.00286815	
0.2	0.00245789	0.00386384	
0.25	0.003007366	0.00526916	Bulk layer: 0.1275184 (a^2)
0.3	0.003595854	0.00814626	Interfacial layer: 0.0720257 (a^2)
0.35	0.004340206	0.01678190	
$T_g^s \approx 0.4$	0.005447014	0.04672654	
$T_g^b \approx 0.45$	0.009241172	0.148054433	

3 COMPUTATION OF <|ΔZ|>, <MSD>, ρ, AND $\Delta\rho$

To understand how the local environment of a bead affected its mobility, we defined four time-dependent quantities: (1) mean absolute Z-displacement (<|Δz|>), (2) mean square displacements (<MSD>, different from the manner in which MSD was defined in the previous section, (3) local number density ρ_N, and (4) local number density gradient $\Delta\rho_N$. These quantities could be calculated for any CG bead at any instant of time and are discussed next.

Consider a discrete time-dependent motion where a bead at two subsequent time steps (t^i) and (t^{i+1}) has position vectors, Z-coordinates, and local number densities as $\overrightarrow{r}^i$ and $\overrightarrow{r}^{i+1}$, z^i and z^{i+1}, and ρ_N^i and ρ_N^{i+1}, respectively. We then define following quantities: gradient in local number density $\Delta\rho_N{}^{i+\frac{1}{2}} = \rho_N{}^{i+1} - \rho_N{}^i$, squared Z-displacement $|\Delta z^{i+\frac{1}{2}}|^2 = (z^{i+1} - z^i)\cdot(z^{i+1} - z^i)$, and the squared displacement $|\Delta\overrightarrow{r}^{i+\frac{1}{2}}|^2 = (\overrightarrow{r}^{i+1} - \overrightarrow{r}^i)\cdot(\overrightarrow{r}^{i+1} - \overrightarrow{r}^i)$. For any bead, at a given time increment from t^i to t^{i+1}, the incremental mobility was characterized in terms of these defined quantities, $|\Delta z^{i+\frac{1}{2}}|^2$, and $|\Delta\overrightarrow{r}^{i+\frac{1}{2}}|^2$, and correlated with $\Delta\rho_N{}^{i+\frac{1}{2}}$, and ρ_N^i. An example of computing the Z-displacement, and gradient in the local number density is shown in

Figure 4. Thus, over the entire data set, for each value of ρ_N and $\Delta\rho_N$, depending on the choice of incremental time step, beads, and duration of motion, we could find the average values of mean squared Z-displacement $<|\Delta z^{i+\frac{1}{2}}|^2>$, and mean squared displacement $<|\Delta r^{i+\frac{1}{2}}|^2>$, and plot the correlations. While the exhaustive data from LAMMPS was collected at every 0.5τ, a time interval of 50τ was appropriate for capturing the local relative motions between polymer segments (as reported in the literature[9]). At 0.5τ interval time resolution only local bead vibrations were being captured.

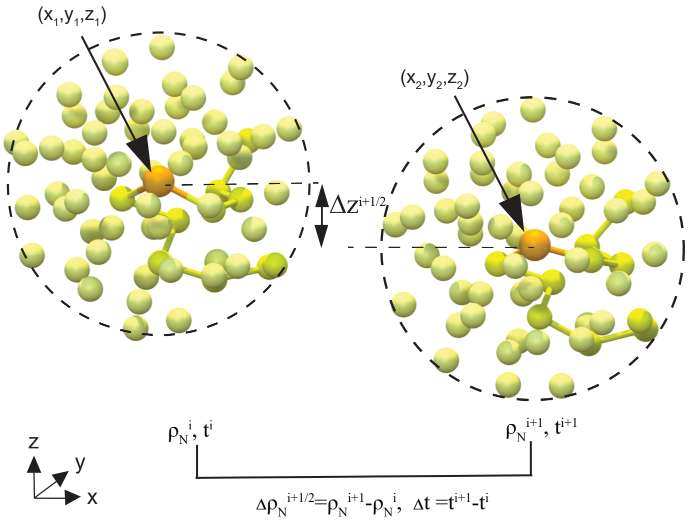

Figure 4. Illustration for computing local number density gradient and associated Z-displacement.

4 DILATATIONAL PLASTICITY

In this section, we discuss the computation of Radial Distribution Function (RDF), and elaborate on the nature of plasticity-mediated enhanced mobility, distribution of local number density, and the growth of interfacial bonding through entanglement formation. Sample results to achieve quick and strong bonding through deformation are also presented.

Calculation of Radial Distribution Function (RDF): The RDF represents the probability $g(r)$ of finding a bead in a shell that is at a distance r from a reference bead. It also captures how closely are the beads packed together in a molecular system. To construct RDF, for any bead in the system, a series of concentric spherical shells are drawn, separated by a fixed distance Δr (see Figure 5), and average number of beads $(n(r))$ in an annular shell region, at distance r and thickness Δr, are computed. Then, to obtain $g(r)$, $n(r)$ is divided by the volume of each annular shell $(4\pi r^2\Delta r)$, and the average particle density ρ_{avg}, where $\rho_{avg} = N/V_{box}$ with N being the total number of particles in the system, and V denoting the system volume. Thus, the RDF is formally given as

$$g(r) = n(r)/(\rho_{avg} * 4\pi r^2\Delta r).$$

In the present analysis, Δr is chosen as $r_{\text{cut-off}}/h$, where $r_{\text{cut-off}} = 3.5a$, and $h = 200$. LAMMPS simulations provide molecular states which are used to construct RDF plots in the commercial post-processing software OVITO.

Table 2 shows a summary of dilatation and densification data collected for a number of chain-ends in the interfacial region during deformation. The chain-ends are labeled according the ID of the chain to which they belong followed by their position in the chain, e.g., chain-end ID 29-1 indicates that this chain-end belongs to the chain with ID 29, and it has a position number 1 in the chain. From the tabulated data, we conclude that number of dilatation events are approximately same as number of densification events, which is consistent with the fact that during deformation RDF plots in deforming glasses are not altered in any noticeable manner.

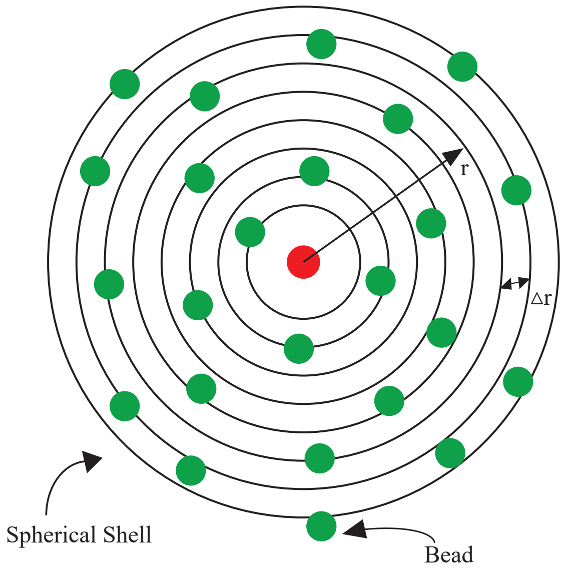

Figure 5. Schematic explaining the calculation of Radial Distribution Function (RDF).

Figure shows 6 distribution of local number density in the “broad zone”, described in the main letter, for equilibrated melt (at T=$0.5u_o/k_B$), undeformed glass (at T=$0.3u_o/k_B$), and deformed glass at 5%, 10%, 15%, 20%, 25%, and 30% plastic strains (at T=$0.3u_o/k_B$). The local number density distribution in the “broad zone” is similar to what was found in the “narrow zone”, i.e., equilibrated melts have lower local number density, and in a deforming glass the local number density changes as deformation proceeds; however, there is no indication of increased liquidity in the deforming glass.

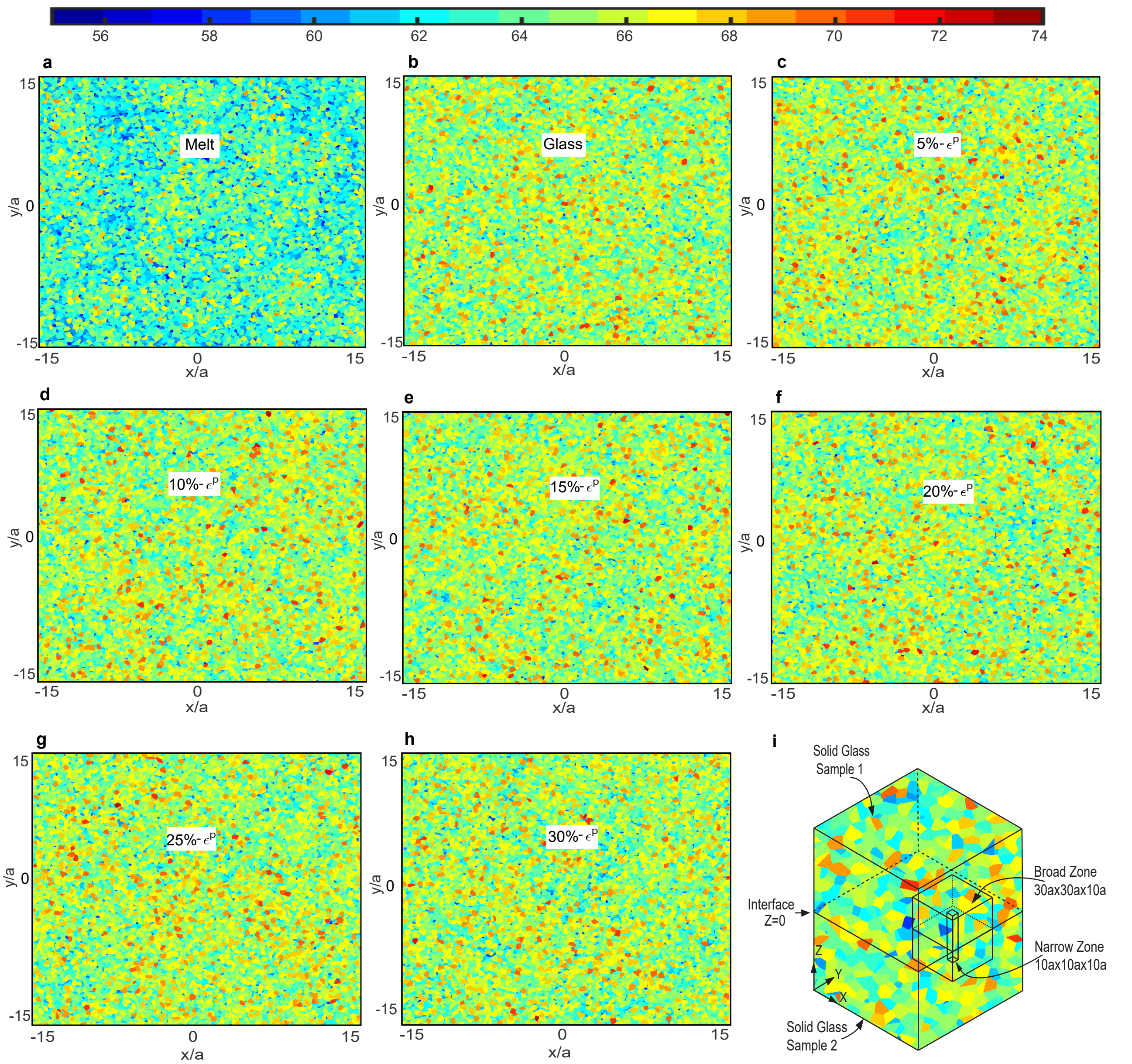

Figure 6. Local number density distribution in the "broad zone" of size $30a \times 30a \times 10a$ near the interface. **a**, Color bar for the local number density. **b-i**, Local number density distribution in the melt at T=0.5u_o/k_B, glass at T=0.3u_o/k_B, and deformed samples at 5%, 10%, 15%, 20% ,25%,& 30% plastic strains, respectively, at T=0.3u_o/k_B. Local number density is calculated by considering 2.5a unit spheres around the beads.

Table 2. Summary of tracking dilatation and densification events, at an interval of 0.5 τ, for chain-ends.

**Chain-end** ID	**Number of dilatational events** ($\Delta\rho_N < 0$)	**Number of volume neutral events** ($\Delta\rho_N = 0$)	**Number of densifications events** ($\Delta\rho_N > 0$)	**Ratio of densifications to dilatations** ($\Delta\rho_N < 0/\Delta\rho_N > 0$)
29-1	1605	710	1586	1.01198
32-500	1573	753	1575	0.99873
222-1	1562	734	1605	0.973209
315-500	1590	725	1586	1.002522
335-500	1587	747	1567	1.012763
420-1	1595	704	1602	0.99563
447-1	1594	722	1585	1.005678
455-500	1555	789	1557	0.998715
465-500	1606	690	1605	1.000623
504-1	1574	717	1610	0.97764
518-1	1597	724	1580	1.010759
565-500	1546	791	1564	0.988491
570-1	1563	775	1563	1
581-1	1632	663	1606	1.016189
595-1	1566	754	1581	0.990512
647-500	1570	723	1608	0.976368
691-500	1559	755	1587	0.982357
701-500	1583	763	1555	1.018006
744-500	1583	761	1557	1.016699
817-1	1544	824	1533	1.007175
870-500	1583	776	1542	1.026589
937-1	1556	772	1573	0.989193
990-500	1583	723	1595	0.992476

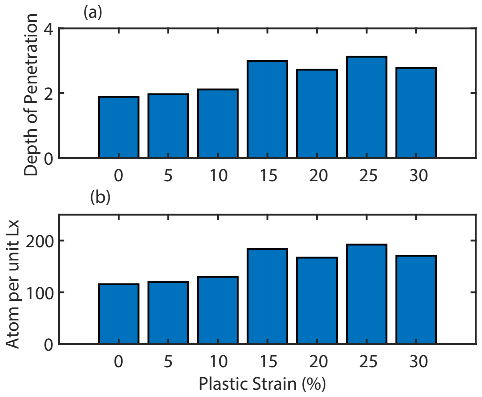

Figure 7. (a) Depth of penetration or interfacial region with respect to plastic deformation, and (b) number of atoms per unit interfacial width Lx (along the X-direction).

Figure 7 shows the growth of the interfacial region as plastic strain is imposed. At 0% plastic strain the two sample boxes under Van der Waals interactions come closer into molecular proximity, even before plastic deformation is imposed, such that, they create a molecular-scale overlap. We refrain from "removing" this overlap from our data. It is seen that as plastic deformation proceeds, the interfacial region expands (in the Z-direction), Figure 7(a) and, new beads per unit interfacial width enter the interfacial region, Figure 7(b). Both trends are non-monotonic. We utilized the Z1-code to study the growth of entanglements near the interface (located at z/a=33.5). As the compression proceeded in the Z-direction, and plane strain conditions are maintained in the Y-direction, the sample expanded in the X-direction, thus, we computed the normalized entanglement density (defined as the total number of entanglements in a units thick layer along the Z-direction, divided by the the length of the bonded interface in the X-direction Lx). The plot of the normalized entanglement density with respect to the Z-dimension, is shown in Figure 8. We note that as the plastic strain is imposed, the normalized entanglement density first increases from 5% to 10% plastic strain, then decreases for 15%, and 20% plastic strains, and then again increases at 25%, and 30% plastic strains. This non-monotonic behavior is consistent with the non-monotonic trends of W_f with respect to plastic strain, both, in our molecular simulations and experiments. To ensure the reproducibility of these non-monotonic trends, we conducted additional simulations and recovered similar trends (see Section 6).

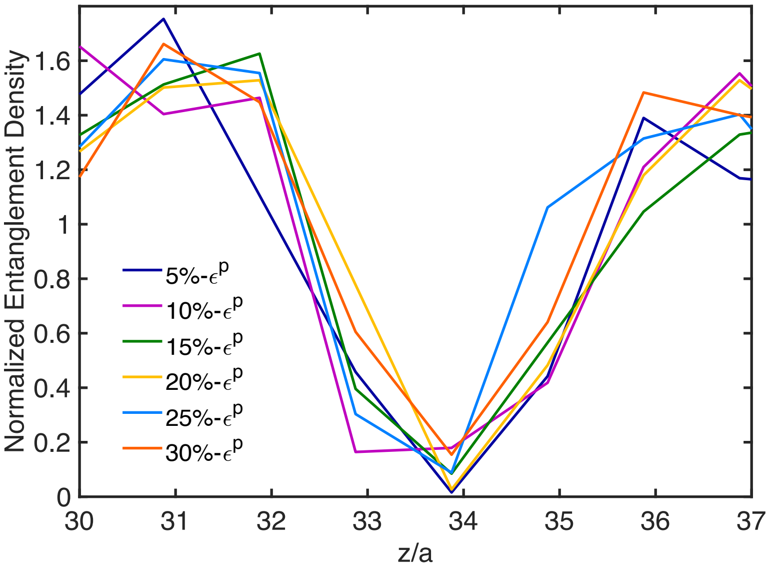

Figure 8. Normalized entanglement density of the deformed samples with respect to height. The bonding interface is located at height of approximately z/a=33.5.

5 COMPARISON OF BONDING STRENGTHS

For the samples considered so far, bonding experiments at T= $0.3u_o/k_B$ revealed that through deformation, ductile strengths only up to 11% of the bulk strength were obtained, as shown in Figure 9. We recollect that the bonding temperature $T = 0.3u_o/k_B$ is significantly lower than the bulk glass-transition temperature ($T_g^b = 0.445u_o/k_B$), and the relatively weak bonding noted is not surprising. To verify that strengths comparable to that of bulk can be rapidly obtained via deformation-induced bonding, we carried out bonding experiments on two different equilibrated samples at $T = 0.42u_o/k_B$ with $N = 100$ and $T_g^b = 0.443u_o/k_B$, and compared them with the bulk strength of the same polymer, Figure 10(a) & (b). This choice of material parameters and processing conditions was motivated by the hypotheses that: (i) lowering the molecular weight increases the number of chain-ends on the free surface of the sample, and we have already seen that chain ends play a critical role in interpenetration and formation of entanglements which yields stronger bonding, and (ii) increasing the temperature will make the polymer matrix more ductile, and deformation will amplify the already existing high-mobility at the free-surface to yield quick bonding. We were able to obtain strong bonding strengths approaching towards the bulk strength. It was noted that at one of the higher plastic strains for N=100 failure occurred in the bulk rather than at the interface (Figure 10(a) for 25% plastic strain, which showed the highest strength). These results indicate that optimization in processing parameters (strain rates and temperatures) and material conditions can yield strong bonding rather quickly via deformation and deformation-acceleration in thermoplastics have now opened a new mechanistic pathway unknown to scientists and practitioners, thus far. It should be noted that the absolute strengths of the sample with $N = 100$ have reduced compared to $N = 500$, however, the fact that we are able to start approaching bulk-like strengths is a remarkable result in its own right. We speculate that bulk strengths can also be achieved for higher molecular weight glasses (like $N = 500$) via deformation quickly (compared to long-time interdiffusion), and will require detailed optimization of processing conditions or even alterations in the loading program. We defer such interesting and potentially transformative explorations for the future.

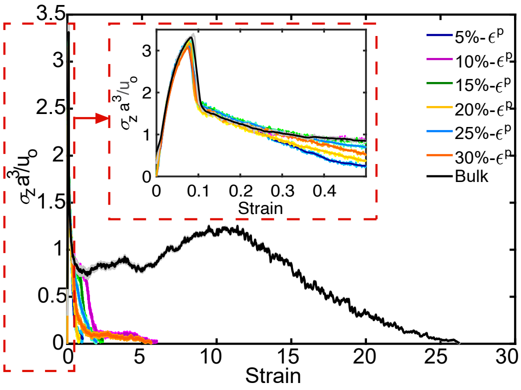

Figure 9. Comparison of bonding strengths with bulk tensile test for samples with $N = 500$.

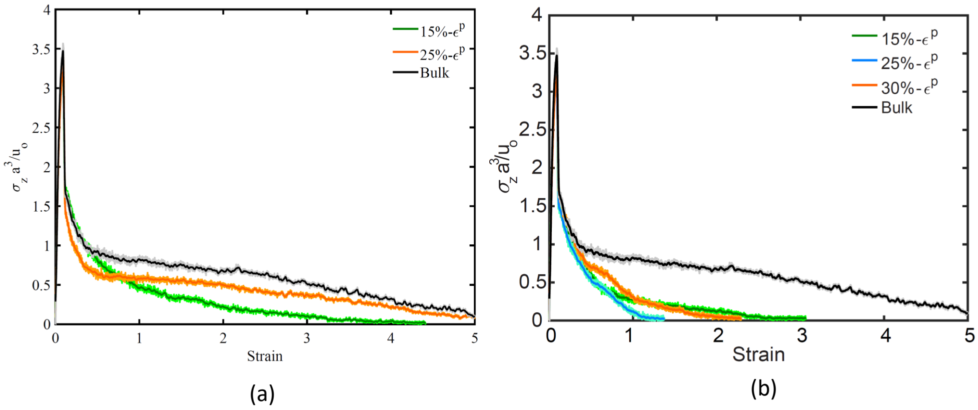

Figure 10. Strong bonding through deformation for two polymer samples with $N = 100$ in (a) and (b).

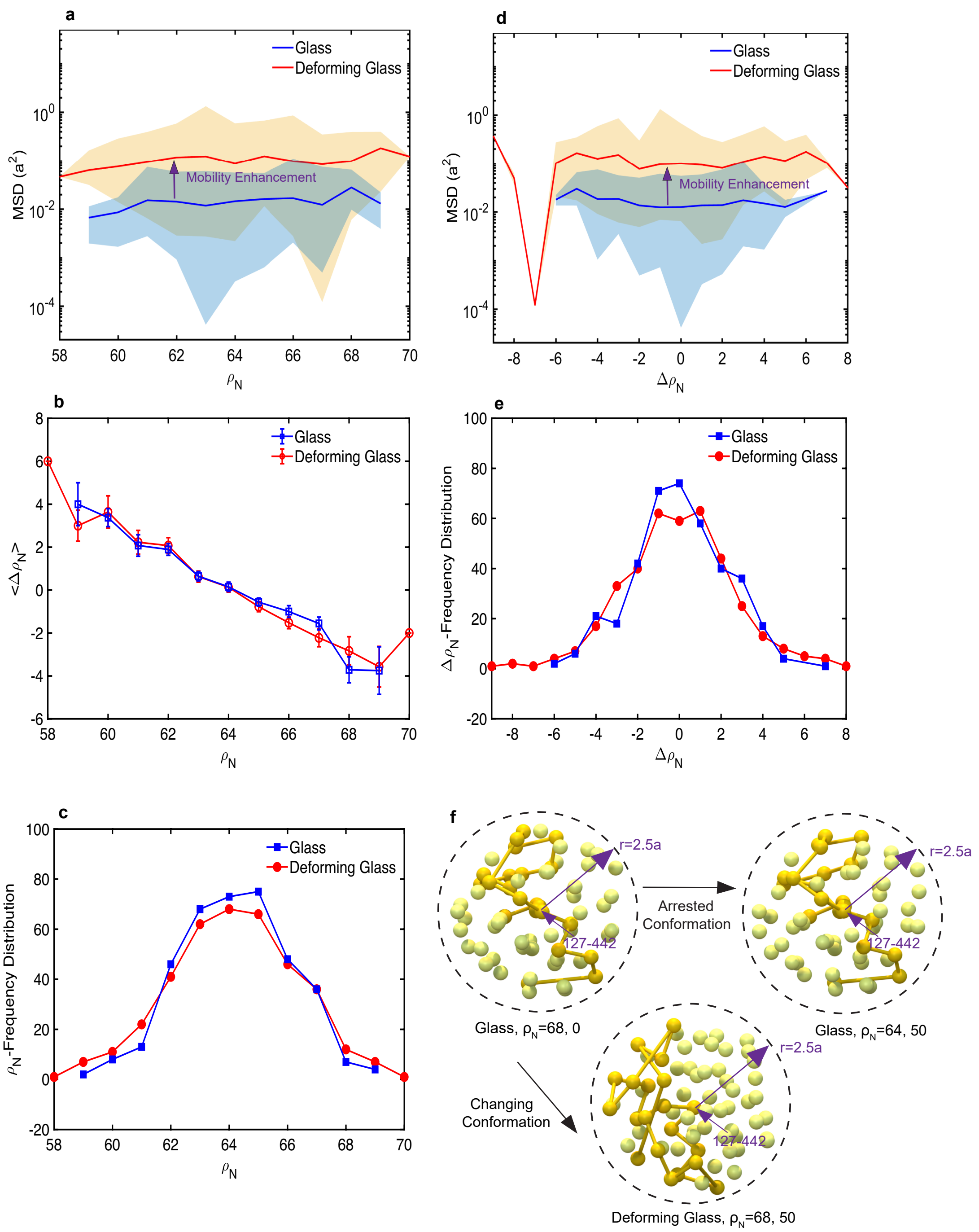

Figure 11. Non-Chain-End-Motion. a, d, Comparison of non-chain-end displacements with respect to local number density, and gradient in local number density. **b**, the correlation between gradient in local number density and local number density. **c**, the frequency distribution of local number density. **e**, the frequency distribution of gradient in local number density. **f**, conformation arrest and transformation in the glassy state and deforming glass, respectively.

6 REPRODUCIBLE NON-MONOTONIC TRENDS

To confirm the reproducibility of the non-monotic bonding trends observed in this study, we prepared two additional glassy samples. We subjected them to deformation-induced bonding at different plastic strains, and debonding tensile failures were carried out. The corresponding work of fracture and normalized entanglement density for the two additional samples (second and third), are shown in Figures 12 and 13, and 14 and 15, respectively. These results are consistent with the non-monotonic trends of the observed bonding with respect to plastic strain.

In the prior experimental results on deformation-induced bonding (DIB)[11, 12], it was comprehensively concluded that molecular interpenetration during bulk plastic deformation across the interface was the mechanism responsible for bonding between glassy interfaces. The experimentally observed trends also exhibited a non-the monotonic trend of work of fracture with respect to plastic strain, i.e., first increasing, then decreasing, and sometimes further increasing. The non-monotonic trends were consistent with the well-known behavior of glassy polymers developing molecular level anisotropy (chain re-orientation) at large plastic strains, such that at increasing plastic strains, there could be re-alignment of the chains parallel to the bonding interface and thereby diminishing the effective entanglement across the interface. These non-monotonic behaviors (for the work of fracture and normalized entanglement density) have been observed repeatedly through the molecular dynamics simulations presented in this paper. We emphasize that the overall trend of the work of fracture, the precise location of the peak work of fracture, will, in general, depend on the material's history, molecular states, and operating conditions (e.g. deformation rates). We defer these for future work and for the broader research community to investigate.

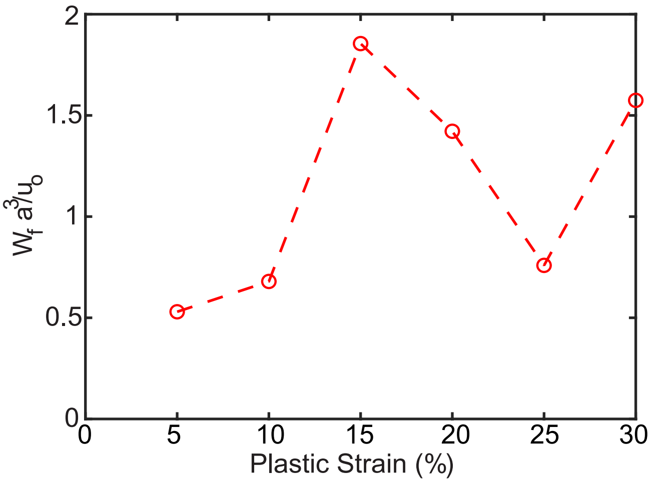

Figure 12. Non-monotonic variation of work of fracture with respect imposed plastic strain for a second randomly prepared sample.

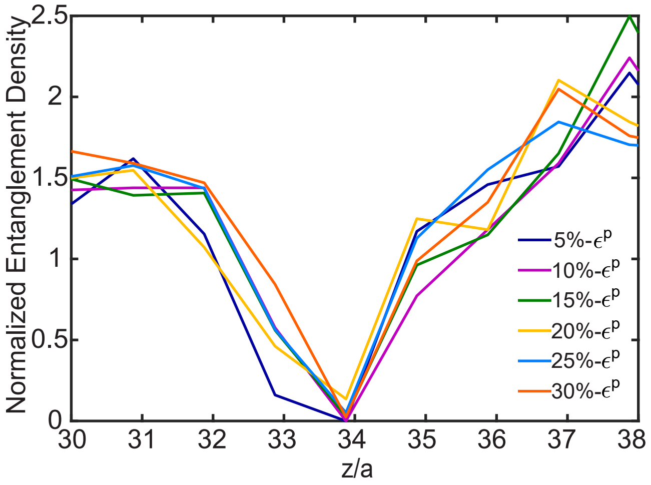

Figure 13. Normalized entanglement density of the deformed samples with respect to height for a second randomly prepared sample. The bonding interface is located at a height of approximately z/a=33.5.

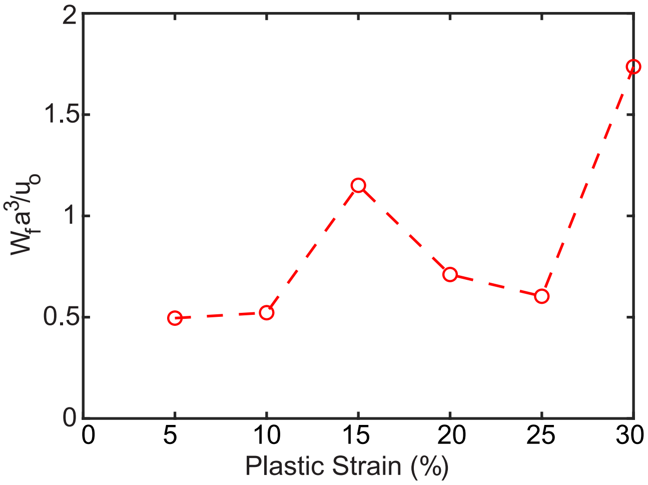

Figure 14. Non-monotonic variation of work of fracture with respect imposed plastic strain for a third randomly prepared sample.

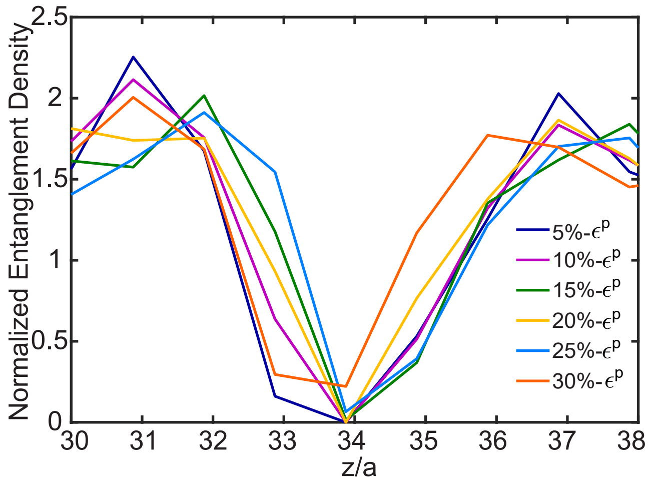

Figure 15. Normalized entanglement density of the deformed samples with respect to height for a third randomly prepared sample. The bonding interface is located at a height of approximately z/a=33.5.

SUPPLEMENTARY VIDEOS

Video 1: Deformation-induced bonding between solid-state polymer samples.

Video 2: Debonding of the deformation-induced bonded interface.

Video 3: Mechanism of bonding through interpenetration of molecular chains across interfaces.

Video 4: Formation of entanglement at the chain-end upon compression, followed by disengagement and reformation of the entanglement.

REFERENCES

1. R. Auhl, R. Everaers, G. S. Grest, K. Kremer, and S. J. Plimpton. Equilibration of long chain polymer melts in computer simulations. *The Journal of chemical physics*, 119(24):12718–12728, 2003.
2. M. Bulacu and E. van der Giessen. Forced reptation revealed by chain pull-out simulations. *The Journal of chemical physics*, 131(6):064904, 2009.
3. M. Bulacu and E. Van der Giessen. Effects of pulling velocity and temperature revealed in polymer pull-out simulations. *EPL (Europhysics Letters)*, 93(6):63001, 2011.
4. T. Ge, G. S. Grest, and M. O. Robbins. Tensile fracture of welded polymer interfaces: Miscibility, entanglements, and crazing. *Macromolecules*, 47(19):6982–6989, 2014.
5. T. Ge, F. Pierce, D. Perahia, G. S. Grest, and M. O. Robbins. Molecular dynamics simulations of polymer welding: Strength from interfacial entanglements. *Physical Review Letters*, 110(9):098301, 2013.
6. T. Ge, M. O. Robbins, D. Perahia, and G. S. Grest. Healing of polymer interfaces: Interfacial dynamics, entanglements, and strength. *Physical Review E*, 90(1):012602, 2014.
7. R. S. Hoy and M. O. Robbins. Strain hardening of polymer glasses: Effect of entanglement density, temperature, and rate. *Journal of Polymer Science Part B: Polymer Physics*, 44(24):3487–3500, 2006.
8. K. Kremer and G. S. Grest. Dynamics of entangled linear polymer melts: A molecular-dynamics simulation. *The Journal of Chemical Physics*, 92(8):5057–5086, 1990.
9. H. Morita, K. Tanaka, T. Kajiyama, T. Nishi, and M. Doi. Study of the glass transition temperature of polymer surface by coarse-grained molecular dynamics simulation. *Macromolecules*, 39(18):6233–6237, 2006.
10. J. T. Padding and W. J. Briels. Time and length scales of polymer melts studied by coarse-grained molecular dynamics simulations. *The Journal of Chemical Physics*, 117(2):925–943, 2002.
11. N. Padhye, D. M. Parks, B. L. Trout, and A. H. Slocum. A new phenomenon: Sub-tg, solid-state, plasticity-induced bonding in polymers. *Scientific Reports*, 7:46405, 2017.
12. N. Padhye and A. Vallabh. Deformation-induced bonding of polymer films below the glass transition temperature. *Journal of Applied Polymer Science*, 138(41):50934, 2021.
13. M. J. Stevens. Interfacial fracture between highly cross-linked polymer networks and a solid surface: effect of interfacial bond density. *Macromolecules*, 34(8):2710–2718, 2001.
